# Elevated Hepatic Copper Content in Porto‐Sinusoidal Vascular Disorder (PSVD): *Leading Down a Wrong Track*

**DOI:** 10.1111/liv.16175

**Published:** 2025-01-14

**Authors:** Lorenz Balcar, Nina Dominik, Behrang Mozayani, Georg Semmler, Emina Halilbasic, Mattias Mandorfer, Thomas Reiberger, Michael Trauner, Bernhard Scheiner, Albert Friedrich Stättermayer

**Affiliations:** ^1^ Division of Gastroenterology and Hepatology, Department of Medicine III Medical University of Vienna Vienna Austria; ^2^ Vienna Hepatic Hemodynamic Lab, Division of Gastroenterology and Hepatology, Department of Medicine III Medical University of Vienna Vienna Austria; ^3^ Clinical Research Group MOTION Medical University of Vienna Vienna Austria; ^4^ Department of Pathology Medical University of Vienna Vienna Austria

**Keywords:** cirrhosis, portal hypertension, PSVD, WD, Wilson disease

## Abstract

**Background and Aims:**

Porto‐sinusoidal vascular disorder (PSVD) is a rare vascular liver disorder characterised by specific histological findings in the absence of cirrhosis, which is poorly understood in terms of pathophysiology. While elevated hepatic copper content serves as diagnostic hallmark in Wilson disease (WD), hepatic copper content has not yet been investigated in PSVD.

**Methods:**

Patients with a verified diagnosis of PSVD at the Medical University of Vienna and available hepatic copper content at the time of diagnosis of PSVD were retrospectively included. Elevated hepatic copper content was correlated with cholestatic changes and WD diagnostics in PSVD and analysed for liver‐related outcomes (first/further hepatic decompensation/liver‐related death).

**Results:**

Overall, 92 patients were included into this study (mean age 49 ± 16; 57% male; median hepatic copper content was 30 [IQR: 18–55] μg/g) of whom 29 (32%) had moderately (≥ 50 μg/g) and 4 (4%) strongly (≥ 250 μg/g) elevated hepatic copper content.

Elevated levels of hepatic copper were associated with younger age in multivariable linear regression analysis. After adjusting for age, decompensation status and albumin, hepatic copper content was significantly associated with the outcome of interest (log, per 10; aHR: 1.60 [95% CI: 1.14–2.25]; *p* = 0.007). A hepatic copper cut‐off at ≥ 90 μg/g identified PSVD patients with considerable risk of liver‐related outcomes (at 2 years: 51% vs. 12%).

**Conclusion:**

Elevated hepatic copper seems frequent in patients with PSVD even in the absence of cholestatic features, especially in young patients, which makes differential diagnosis to WD challenging. Since PSVD patients with elevated hepatic copper content had increased risk for liver‐related outcomes, the pathomechanisms underlying hepatic copper accumulation in PSVD should be investigated as this may open new therapeutic avenues.

**Summary**

- Hepatic copper content is frequently elevated in porto‐sinusoidal vascular disorder
- This is especially pronounced in young patients with and without elevated cholestasis parameters
- Elevated hepatic copper content was associated with worse liver‐related outcomes
- Specific pathomechanisms that may offer therapeutic avenues remain to be investigated in future studies

**Abbreviations**
AP: alkaline phosphatase
ATP7B: adenosin triphosphatase copper‐transporting beta
HVPG: hepatic venous pressure gradient
NRH: nodular regenerative hyperplasia
PSVD: porto‐sinusoidal vascular disorder
WD: Wilson disease

## 1 Introduction

Porto‐sinusoidal vascular disorder (PSVD), a relatively new umbrella term encompassing idiopathic noncirrhotic portal hypertension, is a rare vascular disorder of the liver implementing patients with alterations of the portal and sinusoidal vasculature associated with presinusoidal portal hypertension in the absence of cirrhosis [1, 2]. Typically, severity of portal hypertension stands in contrast with liver stiffness measurement [3, 4] and hepatic venous pressure gradient (HVPG) [3, 4, 5, 6, 7, 8] in the presence of intrahepatic veno‐venous communications [5, 9]. Patients normally present with preserved liver function and might exhibit specific radiomorphological characteristics including less liver surface nodularity, and specific alterations in the periportal area [10, 11].

However, diagnosis and differentiation from alternative liver diseases remains challenging: First, diagnosis of PSVD requires invasive liver biopsy of adequate quality, as well as substantial expertise in liver pathology. Second, they remain asymptomatic for a long time and only present at later stages of disease after portal hypertension‐associated complications have already developed [12]. Currently, noninvasive tests—especially the combination of liver and spleen stiffness measurement—are being increasingly studied for early detection of PSVD [13, 14]. However, no tests or biomarkers have so far been proven to be accurate in diagnosing PSVD.

Copper is an essential trace element that is incorporated in several anti‐inflammatory hepatic enzymes like the copper‐zinc superoxide dismutase or in metallopeptidases like metallothionines. While ingested, a small fraction is absorbed by enterocytes in the duodenum and proximal small intestine and consequently transported in the portal circulation to be avidly removed by the liver. Copper is utilised by hepatocytes for metabolic capacities; incorporated via adenosin triphosphatase copper‐transporting beta (*ATP7B*)—a P‐type glycoprotein residing in the trans‐Golgi network of hepatocytes—which is responsible for incorporation of copper into apo‐ceruloplasmin and secretory biliary pathways [15, 16]. Most excess copper is excreted through this pathway into faeces. Impaired biliary copper excretion leads to hepatic copper retention [17, 18]. Variants in the *ATP7B* gene decreases intracellular transmembrane transport of copper leading to toxic copper accumulation and a disease known as Wilson disease (WD) [15, 16]. Hepatic parenchymal copper concentration ≥ 250 μg/g dry weight provides critical clinical diagnostic information [18, 19], whereas normal concentration (< 50 μg/g) almost entirely excludes diagnosis. Since patients with WD may already present at young age with clinical symptoms of portal hypertension, they may have a similar clinical phenotype compared to PSVD patients. An interesting clinical case of an adolescent patient with suspicion of WD based on elevated hepatic copper level and symptoms of portal hypertension prompted us to systematically investigate copper accumulation in patients with PSVD.

The objective of this study was to evaluate the prevalence of elevated hepatic copper content and to determine the clinical implications of these pathomechanistic changes in patients with PSVD.

## 2 Methods

### 2.1 Patient Cohort and Study Design

**FIGURE 1:**
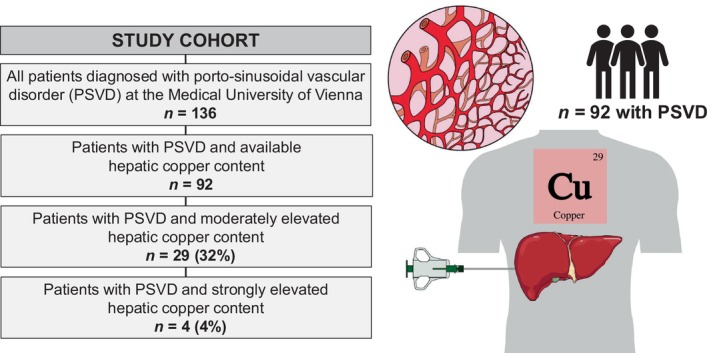
Study description.

One hundred thirty‐six patients with histologically confirmed PSVD participating in the prospective Vienna Vascular Liver Disease Study (VALID study, ClinicalTrials.gov Identifier: NCT03541057) were included in this retrospective, unicentric study. All PSVD patients with available hepatic copper content were included (*n* = 92; Figure 1). Measurement of hepatic copper content has been routinely performed in patients with PSVD and has been included in the work‐up of these patients at our tertiary care centre. Clinical and laboratory data as well as data on liver stiffness measurement were collected at the time of HVPG measurement and (transjugular) liver biopsy. All samples were collected after an overnight fasting period.

### 2.2 Definitions

Diagnosis of PSVD was established applying diagnostic and exclusion criteria as previously described [1]. Specifically, PSVD was diagnosed based on a liver biopsy of adequate quality to rule out cirrhosis in the presence of *either* one clinical sign *specific* for portal hypertension *or* one histological lesion *specific* for PSVD. Specific clinical signs of portal hypertension include the presence of gastroesophageal varices, or portosystemic collaterals on endoscopy or cross‐sectional imaging, or a history of portal‐hypertensive bleeding. Specific histological findings include obliterative portal venopathy, nodular regenerative hyperplasia (NRH), or incomplete septal fibrosis. Alternatively, the diagnosis of PSVD was established in a patient without cirrhosis on liver biopsy presenting with *both* an *unspecific* clinical sign of PH *and* an *unspecific* histological sign for PSVD. Unspecific clinical signs of PH include the presence of ascites, thrombocytopenia and splenomegaly (≥ 13 cm). Unspecific histological signs include portal tract abnormalities, architectural disturbances, nonzonal sinusoidal dilatation and mild perisinusoidal fibrosis.

Cholestasis was defined either pathologically, radiologically by magnetic resonance cholangiopancreatography and/or elevated alkaline phosphatase (AP; ULN ≥ 1.67) and/or bile acids (ULN). Importantly, with this work‐up we tried to exclude other causes of cholestasis and correlate elevated hepatic copper content in the absence of any cholestatic changes.

### 2.3 Genotyping for WD and Copper Diagnostics

PSVD patients with highly elevated hepatic copper content (≥ 250 μg/g) were analysed for presence of variants in *ATP7B*. Genetic analysis were performed as described elsewhere [20]. Routine laboratory tests were obtained by the ISO‐certified Department of Laboratory Medicine of the Medical University of Vienna using commercially available methods that are applied in clinical routine and blood samples obtained via a central venous line (i.e., the side port of the catheter introducer sheath) at the time of HVPG measurement (e.g., ceruloplasmin, serum copper). Hepatic copper content (in μg/g dry weight) was measured by flame atomic absorption spectroscopy according to Kingston and Jassie [21], with an ULN of 50 μg/g dry weight. Patients were stratified by the degree of hepatic copper content (moderately: ≥ 50 μg/g; highly elevated: ≥ 250 μg/g).

### 2.4 Histological Work‐Up

Liver biopsy to adequately diagnose PSVD were required to have a length of ≥ 20 mm and/or include ≥ 6 portal tracts and/or be considered adequate for the exclusion of cirrhosis by an expert liver pathologist [1]. Liver biopsies were evaluated by expert liver pathologists for the presence/absence of cirrhosis or histological features of PSVD (as described before).

### 2.5 Hepatic Venous Pressure Gradient Measurement

HVPG measurements were performed at the Vienna Hepatic Hemodynamic Lab according to a standardised and published protocol in the PSVD and the advanced chronic liver disease cohort [22]. HVPG was calculated as the mean difference between the wedged hepatic vein pressure and the free hepatic vein pressure after three measurements [23]. Transjugular liver biopsies were obtained using an aspiration or the TruCut biopsy set, as previously described [22, 24].

### 2.6 Elastography

Liver stiffness measurement was performed using FibroScan (Echosens, Paris, France) by experienced operators, as previously described [25, 26]. The M and XL probes were used according to the recommendations of the manufacturer.

### 2.7 Statistical Analysis

Statistical analyses were performed using R 4.3.3 (R Core Team, R Foundation for Statistical Computing, Vienna, Austria). Continuous variables were reported as mean ± standard deviation (for variables in Gaussian distribution) or median and interquartile range (IQR; for variables in non‐Gaussian distribution). Categorical variables were reported as absolute (*n*) and relative (%) proportion of patients with/without a certain characteristic. Student's *t*‐test was used for group comparisons of normally distributed variables and Mann–Whitney *U* test for non‐normally distributed variables, respectively. Group comparisons of categorical variables were performed using either Pearson's chi‐squared (*χ* ^2^) or Fisher's exact test, as applicable. Patients were stratified according to copper content as well as according to the presence/absence of cholestasis.

Univariable and multivariable linear regression analyses were applied to evaluate factors associated with hepatic copper content.

Follow‐up time was calculated as the time from diagnosis to the date of liver transplantation, death, or last follow‐up at one of the hospitals of the Vienna hospital association by the reverse Kaplan–Meier method. Impact of hepatic copper content on liver‐related outcomes was assessed using Cox regression analyses. Composite endpoints of interest were chosen according to disease stage; first decompensation or liver‐related death for patients with compensated disease; further decompensation (as defined by Baveno VII [27]), or liver‐related death for decompensated disease. Variables were included as continuous variables and log‐transformed if variables were skewed. Uni‐ and multivariable Cox regression analyses were performed to evaluate parameters independently associated with the events of interest. Multivariable Cox regression models were based on expert opinion including factors associated with outcomes in PSVD patients (i.e., variables that were judged as clinically relevant according to available literature [28]). To determine the most adequate cut‐off for prognostication of the outcome of interest, maximally selected rank statistics [29] were used by applying the maxstat package in R (https://cran.r‐project.org/web/packages/maxstat/maxstat.pdf). For Kaplan–Meier analyses, the first outcome appearing (as defined above) was considered as the outcome of interest, using the log‐rank test for curve comparison.

The level of significance was set at a two‐sided *p* value < 0.05.

### 2.8 Ethics

This study was approved by the ethics committee of the Medical University of Vienna (No. 1928/2017). All patients gave written informed consent to the use of their data and samples.

## 3 Results

### 3.1 Patient Characteristics of the Copper Cohort

From the 92 patients included with PSVD, mean age was 49 ± 16 years and 56% of included patients were male (44% female; Table 1). Seventy patients (76%) had specific clinical (vs. *n* = 87, 95% unspecific) and 59 patients (64%, vs. *n* = 78, 85% unspecific) specific histological signs of PSVD. At PSVD diagnosis, 8 patients (9%) already presented with portal vein thrombosis, while a cavernous, transformed portal vein was present in 14 patients (15%). Twenty‐four subjects (26%) presented with ascites, two patients (2%) with (history of) overt hepatic encephalopathy and 15 patients (16%) with a history of variceal bleeding. Overall, 37 patients (40%) were considered decompensated at baseline. Mean HVPG was 7 ± 5 mmHg (range 1–19 mmHg), mean liver stiffness measurement was 11 ± 7 kPa and mean continuous attenuation parameter was 208 ± 63 dB/m. Detailed information about baseline characteristics are summarised in Table 1.

**TABLE 1:** Detailed patient characteristics at the time of diagnosis of PSVD in patients with available hepatic copper content.

Patient characteristics	Hepatic copper content avail. *n* = 92 (100%)	Hepatic copper content < 50 μg/g *n* = 63 (68%)	Hepatic copper content ≥ 50 μg/g *n* = 29 (32%)	*p* value
Demographical data				
Age, years, mean ± SD	48.9 ± 16.3	50.0 ± 16.1	46.6 ± 16.8	0.354
Body mass index, kg/m^2^	24.9 ± 5.1	25.3 ± 5.5	23.9 ± 3.9	0.221
Sex, *n* (%)				
Male	52 (56%)	34 (54%)	18 (62%)	0.466
Female	40 (44%)	29 (46%)	11 (38%)	
Evidence of portal hypertension				
Varices, *n* (%)^a^	57 (70%)	36 (67%)	21 (75%)	0.437
Splenomegaly, *n* (%)^b^	68 (80%)	44 (76%)	24 (83%)	0.376
Collaterals, *n* (%)^b^	59 (69%)	41 (71%)	18 (86%)	0.441
Portal vein thrombosis, *n* (%)	8 (9%)	5 (8%)	3 (10%)	0.704
Cavernoma, *n* (%)	14 (15%)	10 (16%)	4 (14%)	1.000
Presence of ascites, *n* (%)	24 (26%)	14 (23%)	10 (34%)	0.366
History of/current hepatic encephalopathy, *n* (%)	2 (2%)	1 (2%)	1 (3%)	0.533
History of variceal bleeding, *n* (%)	15 (16%)	10 (16%)	5 (17%)	1.000
HVPG, mmHg, mean ± SD	7 ± 5	7 ± 4	8 ± 5	0.092
Liver stiffness measurement, kPa, mean ± SD	11 ± 7	10 ± 6	11 ± 10	0.546
Continuous attenuation parameter, dB/m, mean ± SD	208 ± 63	217 ± 64	186 ± 57	0.072
Diagnostic criteria of PSVD, *n* (%)				
Specific clinical signs	70 (76%)	47 (75%)	23 (79%)	0.623
Unspecific clinical signs	87 (95%)	58 (92%)	29 (100%)	0.176
Specific histological signs	59 (64%)	42 (67%)	17 (59%)	0.455
Obliterative portal venopathy	4 (4.3%)	2 (3.2%)	2 (6.9%)	0.588
Nodular reg. hyperplasia	26 (28.3%)	16 (25.4%)	10 (34.5%)	0.456
Incomplete septal fibrosis	48 (52.2%)	44 (68.9%)	14 (48.3%)	0.063
Unspecific histological signs	78 (85%)	53 (84%)	25 (86%)	1.000
Potential underlying factors of PSVD, *n* (%)				
HIV	5 (5%)	3 (5%)	2 (7%)	0.649
History of specific medications	37 (40%)	25 (40%)	12 (41%)	0.877
Associated comorbidities	42 (46%)	27 (43%)	15 (52%)	0.428
Genetic disorders	7 (8%)	4 (6%)	3 (10%)	0.674
Prothrombotic disorders	15 (16%)	10 (16%)	5 (17%)	1.000
Copper diagnostics, median (IQR) or mean ± SD				
Hepatic copper content, μg/g	30 (18–55)	21 (15‐31)	71 (57–133)	**< 0.001**
Ceruloplasmin, mg/dL	27 ± 8	26 ± 8	29 ± 7	0.176
Serum copper, μg/dL	111 (83–132)	114 (78–125)	112 (96–150)	0.432
Laboratory parameters, median (IQR) or mean ± SD				
Platelet count, G/L	122 (68–193)	117 (65–191)	138 (80–205)	0.599
Sodium, mmol/L	139 ± 3	139 ± 3	139 ± 3	0.893
Creatinine, mg/dL	0.8 (0.6–0.9)	0.8 (0.6–0.9)	0.7 (0.6–0.9)	0.334
Albumin, g/L	38.6 ± 6.1	39.0 ± 5.5	37.7 ± 7.1	0.343
Bilirubin, mg/dL	0.8 (0.6–1.2)	0.9 (0.6–1.2)	0.7 (0.6–1.2)	0.602
AP, U/L	91 (69–154)	87 (62–142)	135 (81–210)	**0.006**
GGT, U/L	65 (35–137)	54 (28–119)	111 (53–166)	**0.040**
Bile acids, μmol/L	9 (3‐19)	8 (3‐19)	9 (4‐19)	0.287
INR	1.2 ± 0.2	1.2 ± 0.2	1.2 ± 0.2	0.765
von Willebrand factor antigen, %	230 ± 94	225 ± 92	240 ± 100	0.489
AST, U/L	35 (25–53)	33 (23–48)	45 (29–74)	**0.016**
ALT, U/L	32 (22–57)	31 (21–48)	41 (24–84)	0.109
CRP, mg/dL	0.3 (0.1–0.8)	0.2 (0.1–0.6)	0.5 (0.1–1.5)	**0.016**
Ammonia, mmol/L	32 (23‐40)	33 (24‐38)	31 (23–42)	0.970
Hepatic iron content, μg/g	397 (255–783)	382 (244–827)	545 (209–717)	0.841
Transferrin, mg/dL	246 ± 68	248 ± 69	242 ± 67	0.699
Transferrin saturation	16 (10‐30)	16 (9‐31)	20 (12‐28)	0.811
Ferritin, μg/L	80 (28–175)	79 (27–212)	85 (31–152)	1.000

*Note:* Categorical variables were reported as absolute (*n*) and relative frequencies (%), whereas continuous variables as mean ± SD or median (interquartile range [IQR]), as appropriate. Student's *t*‐test was used for group comparisons of normally distributed variables and Mann–Whitney *U* test for non‐normally distributed variables. Group comparisons of categorical variables were performed using either chi‐squared or Fisher's exact test, as appropriate. *p* values in bold denote *p* < 0.05.

Abbreviations: ALT, alanine transaminase; AP, alkaline phosphatase; AST, aspartate transaminase; CRP, C‐reactive protein; GGT, gamma‐glutamyl transferase; HIV, human immunodeficiency virus; HVPG, hepatic venous pressure gradient; INR, international normalised ratio; IQR, interquartile range; *n* number; SD, standard deviation.

^a^ Data available in 89% of study cohort.

^b^ Data available in 92% of study cohort.

### 3.2 Copper Diagnostics

**FIGURE 2:**
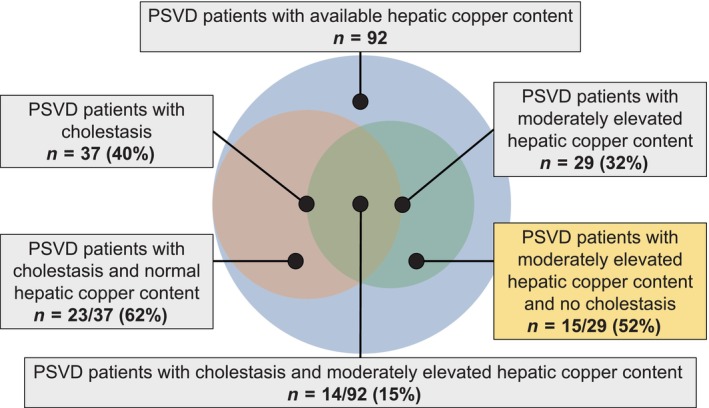
Venn diagram showing the proportion of patients with cholestasis and elevated hepatic copper content and respective intersections.

Median hepatic copper content was 30 (IQR: 18–55; range 3–1136) μg/g. Mean ceruloplasmin was 27 ± 8 mg/dL. Twenty‐nine patients (32%) presented with moderately elevated hepatic copper content (≥ 50 μg/g). Patients with moderately elevated hepatic copper content had higher AP (135 [IQR: 81–210] vs. 87 [IQR: 62–142]; *p* = 0.006), as well as gGT (111 [IQR: 53–166] vs. 54 [IQR: 28–119]; *p* = 0.040) and AST levels (45 [IQR: 29–74] vs. 33 [IQR: 23–48]; *p* = 0.016; Table 1). In those patients, 14 yielded evidence of cholestasis (15%) while 15 did not (16%; Figure 2). Interestingly, patients with moderately elevated hepatic copper content and with vs. without cholestasis were only distinguishable by ceruloplasmin levels (absence of: 25 ± 5 vs. evidence of cholestasis: 33 ± 5; *p* < 0.001), as well as subclinical differences in creatinine, albumin levels and cholestasis parameters (Table 2).

**TABLE 2:** Comparing patient characteristics at the time of diagnosis of PSVD with moderately elevated hepatic copper content (≥ 50 μg/g) with versus without cholestasis.

Patient characteristics	Hepatic copper content ≥ 50 μg/g *n* = 29 (100%)	Hepatic copper content ≥ 50 μg/g with cholestasis *n* = 14 (48%)	Hepatic copper content ≥ 50 μg/g w/o cholestasis *n* = 15 (52%)	*p* value
Demographical data				
Age, years, mean ± SD	46.6 ± 16.8	45.1 ± 12.8	47.8 ± 20.2	0.652
Body mass index, kg/m^2^	23.9 ± 3.9	23.9 ± 3.8	23.9 ± 4.1	0.956
Sex, *n* (%)				
Male	18 (62%)	7 (50%)	11 (73%)	0.196
Female	11 (38%)	7 (50%)	4 (27%)	
Evidence of portal hypertension				
Varices, *n* (%)^a^	21 (75%)	10 (71%)	11 (79%)	1.000
Splenomegaly, *n* (%)^b^	24 (83%)	12 (86%)	12 (93%)	0.861
Collaterals, *n* (%)^b^	18 (86%)	9 (64%)	9 (87%)	0.947
Portal vein thrombosis, *n* (%)	3 (10%)	2 (14%)	1 (7%)	0.598
Cavernoma, *n* (%)	4 (14%)	2 (14%)	2 (13%)	1.000
Presence of ascites, *n* (%)	10 (34%)	5 (36%)	5 (33%)	0.781
History of/current hepatic encephalopathy, *n* (%)	1 (3%)	1 (7%)	—	0.483
History of variceal bleeding, *n* (%)	5 (17%)	2 (14%)	3 (20%)	1.000
HVPG, mmHg, mean ± SD	8 ± 5	9 ± 5	8 ± 5	0.719
Liver stiffness measurement, kPa, mean ± SD	11 ± 10	10 ± 7	13 ± 13	0.454
Continuous attenuation parameter, dB/m, mean ± SD	186 ± 57	163 ± 62	213 ± 38	0.053
Diagnostic criteria of PSVD, *n* (%)				
Specific clinical signs	23 (79%)	11 (79%)	12 (80%)	1.000
Unspecific clinical signs	29 (100%)	14 (100%)	15 (100%)	1.000
Specific histological signs	17 (59%)	7 (50%)	10 (67%)	0.362
Unspecific histological signs	25 (86%)	12 (86%)	13 (87%)	1.000
Potential underlying factors, *n* (%)				
HIV	2 (7%)	1 (7%)	1 (7%)	1.000
History of specific medications	12 (41%)	6 (43%)	6 (40%)	0.876
Associated comorbidities	15 (52%)	9 (64%)	6 (40%)	0.191
Genetic disorders	3 (10%)	3 (21%)	—	0.100
Prothrombotic disorders	5 (17%)	2 (14%)	3 (20%)	1.000
Copper diagnostics, median (IQR) or mean ± SD				
Hepatic copper content, μg/g	71 (57–133)	71 (53–165)	79 (58–142)	0.914
Ceruloplasmin, mg/dL	29 ± 7	33 ± 5	25 ± 5	**< 0.001**
Serum copper, μg/dL	112 (96–150)	140 (101–160)	110 (90–144)	0.354
Laboratory parameters, median (IQR) or mean ± SD				
Platelet count, G/L	138 (80–205)	151 (96–254)	132 (65–181)	0.112
Sodium, mmol/L	139 ± 3	139 ± 3	139 ± 2	0.405
Creatinine, mg/dL	0.7 (0.6–0.9)	0.6 (0.5–0.8)	0.8 (0.6–1.2)	**0.023**
Albumin, g/L	37.7 ± 7.1	34.9 ± 7.4	40.3 ± 5.9	**0.040**
Bilirubin, mg/dL	0.7 (0.6–1.2)	0.7 (0.6–1.2)	0.7 (0.5–1.5)	0.533
AP, U/L	135 (81–210)	210 (133–334)	102 (76–138)	**0.003**
GGT, U/L	111 (53–166)	116 (87–185)	58 (40–157)	0.186
Bile acids, μmol/L	9 (4‐19)	18 (13–73)	5 (2‐9)	**< 0.001**
INR	1.2 ± 0.2	1.2 ± 0.2	1.2 ± 0.3	0.506
von Willebrand factor antigen, %	240 ± 100	261 ± 123	218 ± 68	0.271
AST, U/L	45 (29–74)	49 (28–93)	41 (29–69)	0.747
ALT, U/L	41 (24–84)	42 (24–83)	37 (23–93)	1.000
CRP, mg/dL	0.5 (0.1–1.5)	0.6 (0.2–3.3)	0.5 (0.1–1.0)	0.451
Ammonia, mmol/L	31 (23–42)	34 (25–44)	28 (23‐36)	0.347
Hepatic iron content, μg/g	545 (209–717)	554 (281–679)	468 (171–749)	0.685
Transferrin, mg/dL	242 ± 67	244 ± 75	240 ± 60	0.896
Transferrin saturation	20 (12‐28)	12 (6‐31)	21 (16‐27)	0.252
Ferritin, μg/L	85 (31–152)	77 (18–152)	85 (57–166)	0.462

*Note:* Categorical variables were reported as absolute (*n*) and relative frequencies (%), whereas continuous variables as mean ± SD or median (interquartile range [IQR]), as appropriate. Student's *t*‐test was used for group comparisons of normally distributed variables and Mann–Whitney *U* test for non‐normally distributed variables. Group comparisons of categorical variables were performed using either chi‐squared or Fisher's exact test, as appropriate. *p* values in bold denote *p* < 0.05.

Abbreviations: ALT, alanine transaminase; AP, alkaline phosphatase; AST, aspartate transaminase; CRP, C‐reactive protein; GGT, gamma‐glutamyl transferase; HIV, human immunodeficiency virus; HVPG, hepatic venous pressure gradient; INR, international normalised ratio; IQR, interquartile range; *n*, number; SD, standard deviation.

^a^ Data available in 89% of study cohort.

^b^ Data available in 92% of study cohort.

Elevated levels of hepatic copper content were associated with younger age in multivariable linear regression analysis (Table 3).

**TABLE 3:** Simple and multiple linear regression analysis of factors associated with hepatic copper content.

Patient characteristics	Univariable		Multivariable model	
	*B*	*p* value	*B*	*p* value
Age, per year	−2.470	**0.008**	−2.282	**0.014**
Male vs. female sex	32.921	0.284	—	—
BMI, per kg × m^−2^	−2.758	0.362	—	—
UNOS MELD (2016), per point	−3.770	0.387	—	—
Presence of NRH vs. absence	29.790	0.379	—	—
Ceruloplasmin, per mg × dL^−1^	1.387	0.555	—	—
Bilirubin, per mg × dL^−1^	−3.516	0.770	—	—
AP, per U × L^−1^	0.050	0.619	—	—
GGT, per U × L^−1^	0.015	0.811	—	—
AST, per U × L^−1^	0.289	0.372		
ALT, per U × L^−1^	0.443	0.110	0.328	0.230
Any evidence of cholestasis vs. absence	15.870	0.610	—	—

*Note: p* values in bold denote *p* < 0.05. UNOS MELD (2016) score, United Network for Organ Sharing model for end‐stage liver disease (2016) score.

Abbreviations: ALT, alanine transaminase; AP, alkaline phosphatase; BMI, body mass index; GGT, gamma‐glutamyl transferase; NRH, nodular regenerative hyperplasia.

Overall, four patients (4%) presented with highly elevated hepatic copper content (289 μg/g, 367 μg/g, 745 μg/g, and 1136 μg/g). Three patients were male (all younger than 25 years old), one female (48 years). All had varices at endoscopy (2 small and 2 large varices). Ceruloplasmin levels were normal despite in one patient being minimally below the lower threshold (18.9 mg/dL). Genetic testing for WD identified no pathogenic variants in *ATP7B*. Despite the female patient, none of the other patients showed histological and radiological signs of cholestasis. Urinary copper excretion was lower than 100 μg/24 h in all patients. No patient presented with Kayser–Fleischer rings. Liver biopsy was performed for the work‐up of chronic liver disease with evidence of portal hypertension. All of them had specific histological and clinical signs of PSVD (*n* = 2 with NRH, *n* = 2 with incomplete septal fibrosis). As underlying factor for PSVD, 1 patient had an inflammatory bowel disease with accompanied medication, 1 patient a genetic predisposition and in the other 1 no underlying factors could be detected. All patients presented with severe portal hypertension. One patient had a history of variceal bleeding prior to diagnosis, with refractory bleeding 6 months after diagnosis. Another patient developed ascites 7 months after the diagnosis. All patients were alive at the end of follow‐up.

### 3.3 Outcome Analyses of the Copper Cohort

During a median follow‐up of 3.6 years, 21 (23%) patients developed one or more liver‐related complications (31 events). One patient received liver transplantation, while 6 patients received transjugular intrahepatic portosystemic shunt placement during follow‐up. After adjusting for age, decompensation status, and albumin, hepatic copper content was significantly associated with the outcome of interest in the multistate Cox regression analysis (log, per 10; adjusted hazard ratio [aHR]: 1.60 [95% CI: 1.14–2.25]; *p* = 0.007; Table 4).

**TABLE 4:** Uni‐ and multivariable Cox regression analyses of factors associated with first/further hepatic decompensation and liver‐related death (*n* = 31 events).

Patient characteristics	Univariable		Multivariable	
	HR (95% CI)	*p* value	aHR (95% CI)	*p* value
Age, year, log	2.09 (0.75–5.81)	0.157	2.73 (0.68–11.01)	0.158
History of decompensation	4.79 (2.03–11.30)	**< 0.001**	3.52 (1.43–8.69)	**0.006**
Albumin, log	0.20 (0.05–0.74)	**0.016**	0.70 (0.17–2.87)	0.619
Bilirubin, log	0.85 (0.36–1.99)	0.711	—	—
CRP, log	1.29 (0.99–1.67)	0.055	—	—
Any cholestasis	1.08 (0.50–2.35)	0.841	—	—
Hepatic copper content, per 10, log	1.52 (1.17–1.97)	**0.002**	1.60 (1.14–2.25)	**0.007**

*Note:* *p* values in bold denote *p* < 0.05.

Abbreviations: CRP, C‐reactive protein.

**FIGURE 3:**
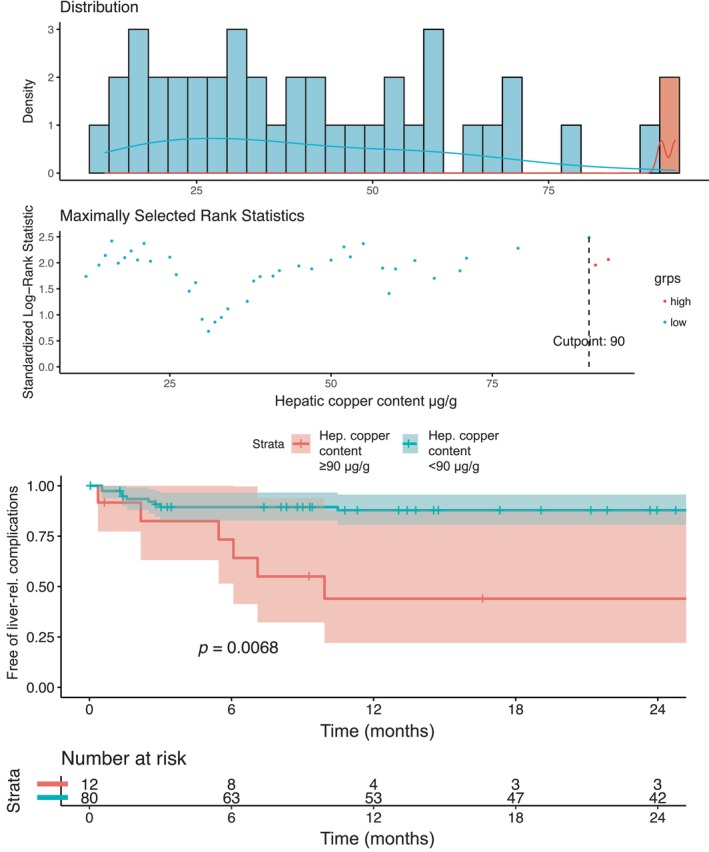
Determination of the most adequate cut‐off assessing hepatic copper content for prognostication of first/further hepatic decompensation/liver‐related death using the maxstat package and stratifying patients according to the respective cut‐off in a Kaplan–Meier curve (hepatic copper content ≥ vs. < 90 μg/g).

A hepatic copper cut‐off at ≥ 90 μg/g identified patients with considerable risk of developing liver‐related outcomes during follow‐up (at 2 years: 51% vs. 12%; *p* = 0.007; Figure 3).

### 3.4 Cholestasis in PSVD Patients

Forty‐six patients (34%) presented with biochemical evidence of cholestasis at PSVD diagnosis. These patients were more frequently female (46% vs. 27%; *p* = 0.001), less frequently associated with HIV as risk factor for PSVD diagnosis (2% vs. 16%; *p* = 0.018), yielded higher ceruloplasmin levels (30 ± 8 vs. 26 ± 7 mg/dL; *p* = 0.014) as well as higher cholestasis parameters (Table S1). Evidence of biochemical cholestasis was not associated with worse prognosis (HR: 1.45 [95% CI: 0.76–2.79]; *p* = 0.261).

## 4 Discussion

This is the first study to show that hepatic copper content is frequently elevated in patients with PSVD. Elevated hepatic copper levels, especially those that are highly elevated, can complicate the diagnosis of PSVD, potentially increasing the risk of misdiagnosis as WD. Importantly, our findings suggest that elevated hepatic copper levels are associated with a worse prognosis in PSVD patients, highlighting the clinical relevance of this observation.

Approximately one‐third of patients with PSVD in our study exhibited moderately elevated hepatic copper levels. This may be related to cholestasis, as hepatic copper accumulation is a known consequence of impaired biliary excretion in various cholestatic conditions, such as primary biliary cholangitis, primary sclerosing cholangitis, familial cholestatic syndromes and cystic fibrosis [30, 31, 32]. Hepatic uptake of dietary copper is not saturable which is why hepatic copper accumulation may easily occur through disturbances in the main mechanisms of hepatobiliary excretion of copper; lysosomal exocytosis via the canalicular membrane process requiring the WD gene product *ATP7B* [31, 33, 34]. In addition, although mutations of hepatic transporter genes are rare, they might exist in heterozygous forms or with incomplete penetrance in a much larger number of individuals, and they may not become evident until the individual is challenged with a condition such as PSVD [31]. There may also be a multimodal pathophysiology explaining the elevated hepatic copper content (with additional genetic [35, 36] and environmental cofactors), also because PSVD is a heterogenic disease entity. In line, almost half of PSVD patients with elevated hepatic copper levels in our study had cholestasis, supporting this potential association. A recent study using metabolomics has shown that there are distinct metabolic signatures in PSVD patients as compared to cirrhotic patients and healthy controls, including alterations in the pyrimidine, glycine, serine and threonine pathways [37]. Among the 283 metabolites tested, 3 bile acids were included, which also emphasises that some form of cholestatic liver injury may be a cause or consequence of PSVD.

In our cohort, PSVD patients with excessively high hepatic copper levels demonstrated a rapid disease progression, suggesting that copper accumulation may contribute directly to disease severity. The exact mechanisms behind this are unclear, but it is hypothesised that excessive copper could exacerbate liver damage through oxidative stress and inactivation of critical cellular functions [38].

Copper distribution within the liver is known to be uneven [39], particularly in conditions like cirrhosis or in regeneration nodes [40], which may affect the accuracy of small tissue samples in reflecting total hepatic copper content. This variability may partly explain discrepancies in our findings. However, the toxicity of copper also depends on its molecular association and subcellular localisation rather than its (total) hepatic concentration.

Importantly, hepatic copper content was significantly associated with liver‐related complications in our cohort. Since we adjusted our model to important covariables, there might be specific disease‐driving pathomechanisms promoting liver‐related complications triggered by elevated hepatic copper content. We can only speculate about potential treatment benefits aiming at lowering the hepatic copper content in PSVD patients.

Due to the nature of this rare disease, we have to acknowledge that the sample size of the PSVD cohort is rather low, especially when considering patients with elevated hepatic copper content. Since this was a retrospective study, we also have to acknowledge all the limitations coming with this study setting. With the broad definition of cholestasis, there might have been false positive diagnoses of ‘cholestatic PSVD patients’ (e.g., elevated bile acids due to porto‐systemic shunting). However, it was important for us to correlate elevated hepatic copper content with PSVD in the absence of any cholestatic changes. Also, we could not provide data explaining pathophysiological mechanisms underlying the higher copper levels in PSVD livers.

In conclusion, this is the first study linking hepatic copper accumulation to PSVD. Elevated hepatic copper levels may be frequently observed in patients with PSVD even in the absence of cholestasis and are associated with an increased risk of liver‐related complications. These findings raise important questions about the underlying mechanisms of copper accumulation in PSVD and its potential role in disease progression, suggesting the need for further investigation that may open new therapeutic avenues.

## Author Contributions

Concept of the study: L.B., A.F.S. Data collection: L.B., N.D., G.S., A.F.S. Statistical analysis: L.B., N.D. Drafting of the manuscript: L.B., N.D., A.F.S. Revision for important intellectual content and approval of the final manuscript: all authors.

## Conflicts of Interest

The authors have nothing to disclose regarding the work under consideration for publication. The following authors disclose conflicts of interests outside the submitted work: L.B. has nothing to disclose. N.D. has nothing to disclose. B.M. has received speakers fees from Astellas Pharma and Springer Medicine. G.S. has nothing to disclose. C.W. has nothing to disclose. M.M. served as a speaker and/or consultant and/or advisory board member for AbbVie, Gilead, Collective Acumen, and W. L. Gore & Associates, Takeda and received travel support from AbbVie, Bristol‐Myers Squibb and Gilead. T.R. served as a speaker and/or consultant and/or advisory board member for AbbVie, Bayer, Boehringer Ingelheim, Gilead, Intercept, MSD, Roche, Siemens and W. L. Gore & Associates and received grants/research support from AbbVie, Boehringer Ingelheim, Gilead, MSD, Philips and W. L. Gore & Associates as well as travel support from Boehringer Ingelheim and Gilead. M.T. received speaker fees from BMS, Falk Foundation, Gilead, Intercept, Ipsen, Jannsen, Madrigal, MSD and Roche; he advised for AbbVie, Albireo, BiomX, Boehringer Ingelheim, Cymabay, Falk Pharma GmbH, Genfit, Gilead, Hightide, Intercept, Ipsen, Janssen, MSD, Novartis, Phenex, Pliant, Rectify, Regulus, Siemens and Shire. He further received travel support from AbbVie, Falk, Gilead, Intercept, and Jannsen and research grants from Albireo, Alnylam, Cymabay, Falk, Gilead, Intercept, MSD, Takeda and UltraGenyx. He is also a co‐inventor of patents on the medical use of norUDCA filed by the Medical Universities of Graz and Vienna. B.S. received travel support from AbbVie, Ipsen and Gilead. A.F.S. has nothing to disclose.

## Supporting information


Data S1.

## Data Availability

The data that support the findings of this study are available from the corresponding author upon reasonable request.

## References

[1] A. De Gottardi , P. E. Rautou , J. Schouten , et al., “Porto‐Sinusoidal Vascular Disease: Proposal and Description of a Novel Entity,” Lancet Gastroenterology & Hepatology 4, no. 5 (2019): 399–411. 30957754 10.1016/S2468-1253(19)30047-0

[2] A. De Gottardi , C. Sempoux , and A. Berzigotti , “Porto‐sinusoidal vascular disorder,” Journal of Hepatology 77, no. 4 (2022): 1124–1135. 35690264 10.1016/j.jhep.2022.05.033

[3] S. Seijo , E. Reverter , R. Miquel , et al., “Role of Hepatic Vein Catheterisation and Transient Elastography in the Diagnosis of Idiopathic Portal Hypertension,” Digestive and Liver Disease: Official Journal of the Italian Society of Gastroenterology and the Italian Association for the Study of the Liver 44, no. 10 (2012): 855–860. 22721839 10.1016/j.dld.2012.05.005

[4] P. Sharma , R. Agarwal , S. Dhawan , et al., “Transient Elastography (Fibroscan) in Patients With Non‐Cirrhotic Portal Fibrosis,” Journal of Clinical and Experimental Hepatology 7, no. 3 (2017): 230–234. 28970710 10.1016/j.jceh.2017.03.002 PMC5620350

[5] R. Vuppalanchi , K. Mathur , M. Pyko , N. Samala , and N. Chalasani , “Liver Stiffness Measurements in Patients With Noncirrhotic Portal Hypertension‐The Devil Is in the Details,” Hepatology (Baltimore, Md) 68, no. 6 (2018): 2438–2440. 10.1002/hep.30167 30014586

[6] J. Bissonnette , A. Généreux , J. Côté , et al., “Hepatic Hemodynamics in 24 Patients With Nodular Regenerative Hyperplasia and Symptomatic Portal Hypertension,” Journal of Gastroenterology and Hepatology 27, no. 8 (2012): 1336–1340. 22554152 10.1111/j.1440-1746.2012.07168.x

[7] B. L. Da , P. Surana , D. Kapuria , et al., “Portal Pressure in Non‐Cirrhotic Portal Hypertension: To Measure or Not to Measure,” Hepatology (Baltimore, Md) 70 (2019): 2228–2230. 10.1002/hep.30862 PMC8191387 31318454

[8] K. Wöran , G. Semmler , M. Jachs , et al., “Clinical Course of Porto‐Sinusoidal Vascular Disease (PSVD) is Distinct From Idiopathic Non‐Cirrhotic Portal Hypertension (INCPH),” Clinical Gastroenterology and Hepatology: The Official Clinical Practice Journal of the American Gastroenterological Association 20, no. 2 (2020): e251–e266. 33279774 10.1016/j.cgh.2020.11.039

[9] S. Futagawa , M. Fukazawa , H. Musha , et al., “Hepatic Venography in Noncirrhotic Idiopathic Portal Hypertension. Comparison With Cirrhosis of the Liver,” Radiology 141, no. 2 (1981): 303–309. 7291551 10.1148/radiology.141.2.7291551

[10] K. Lampichler , G. Semmler , K. Wöran , et al., “Imaging Features Facilitate Diagnosis of Porto‐Sinusoidal Vascular Disorder,” European Radiology 33 (2022): 1422–1432. 36166087 10.1007/s00330-022-09132-4 PMC9889423

[11] S. R. Valainathan , R. Sartoris , L. Elkrief , et al., “Contrast‐Enhanced CT and Liver Surface Nodularity for the Diagnosis of Porto‐Sinusoidal Vascular Disorder: A Case‐Control Study,” Hepatology (Baltimore, Md) 76 (2022): 418–428. 10.1002/hep.32367 PMC9544289 35092315

[12] K. Wöran , G. Semmler , M. Jachs , et al., “Clinical Course of Porto‐Sinusoidal Vascular Disease Is Distinct From Idiopathic Noncirrhotic Portal Hypertension,” Clinical Gastroenterology and Hepatology 20, no. 2 (2022): e251–e266. 33279774 10.1016/j.cgh.2020.11.039

[13] Y. Furuichi , F. Moriyasu , J. Taira , et al., “Noninvasive Diagnostic Method for Idiopathic Portal Hypertension Based on Measurements of Liver and Spleen Stiffness by ARFI Elastography,” Journal of Gastroenterology 48, no. 9 (2013): 1061–1068. 23142969 10.1007/s00535-012-0703-z

[14] J. Ferreira‐Silva , R. Gaspar , R. Liberal , H. Cardoso , and G. Macedo , “Splenic‐Hepatic Elastography Index Is Useful in Differentiating Between Porto‐Sinusoidal Vascular Disease and Cirrhosis in Patients With Portal Hypertension,” Digestive and Liver Disease 55 (2022): 75–80. 36280435 10.1016/j.dld.2022.09.018

[15] S. Lutsenko and M. J. Petris , “Function and Regulation of the Mammalian Copper‐Transporting ATPases: Insights From Biochemical and Cell Biological Approaches,” Journal of Membrane Biology 191, no. 1 (2003): 1–12. 12532272 10.1007/s00232-002-1040-6

[16] T. Y. Tao and J. D. Gitlin , “Hepatic Copper Metabolism: Insights From Genetic Disease,” Hepatology 37, no. 6 (2003): 1241–1247. 12773998 10.1053/jhep.2003.50281

[17] J. D. Gitlin , “Wilson Disease,” Gastroenterology 125, no. 6 (2003): 1868–1877. 14724838 10.1053/j.gastro.2003.05.010

[18] M. L. Schilsky , E. A. Roberts , J. M. Bronstein , et al., “A Multidisciplinary Approach to the Diagnosis and Management of Wilson Disease: Executive Summary of the 2022 Practice Guidance on Wilson Disease From the American Association for the Study of Liver Diseases,” Hepatology 77, no. 4 (2023): 1428–1455. 36152019 10.1002/hep.32805

[19] J. Ludwig , T. P. Moyer , and J. Rakela , “The Liver Biopsy Diagnosis of Wilson's Disease. Methods in Pathology,” American Journal of Clinical Pathology 102, no. 4 (1994): 443–446. 7942601 10.1093/ajcp/102.4.443

[20] P. Ferenci , W. Stremmel , A. Członkowska , et al., “Age and Sex but Not ATP7B Genotype Effectively Influence the Clinical Phenotype of Wilson Disease,” Hepatology 69, no. 4 (2019): 1464–1476. 30232804 10.1002/hep.30280

[21] H. M. Kingston and L. B. Jassie , “Microwave Energy for Acid Decomposition at Elevated Temperatures and Pressures Using Biological and Botanical Samples,” Analytical Chemistry 58, no. 12 (1986): 2534–2541. 3789404 10.1021/ac00125a038

[22] T. Reiberger , P. Schwabl , M. Trauner , et al., “Measurement of the Hepatic Venous Pressure Gradient and Transjugular Liver Biopsy,” JoVE 160 (2020): e58819. 10.3791/58819 32628153

[23] T. Reiberger , A. Ferlitsch , B. A. Payer , et al., “Non‐selective Beta‐Blockers Improve the Correlation of Liver Stiffness and Portal Pressure in Advanced Cirrhosis,” Journal of Gastroenterology 47, no. 5 (2012): 561–568. 22170417 10.1007/s00535-011-0517-4

[24] J. Stift , G. Semmler , C. Walzel , et al., “Transjugular Aspiration Liver Biopsy Performed by Hepatologists Trained in HVPG Measurements Is Safe and Provides Important Diagnostic Information,” Digestive and Liver Disease 51, no. 8 (2019): 1144–1151. 30862438 10.1016/j.dld.2019.01.020

[25] T. Reiberger , A. Ferlitsch , B. A. Payer , et al., “Noninvasive Screening for Liver Fibrosis and Portal Hypertension by Transient Elastography—A Large Single Center Experience,” Wiener Klinische Wochenschrift 124, no. 11 (2012): 395–402. 22699260 10.1007/s00508-012-0190-5

[26] G. Semmler , K. Wöran , B. Scheiner , et al., “Novel Reliability Criteria for Controlled Attenuation Parameter Assessments for Non‐Invasive Evaluation of Hepatic Steatosis,” United European Gastroenterology Journal 8, no. 3 (2020): 321–331. 32213023 10.1177/2050640619900820 PMC7184665

[27] R. de Franchis , J. Bosch , G. Garcia‐Tsao , et al., “Baveno VII—Renewing Consensus in Portal Hypertension,” Journal of Hepatology 76, no. 4 (2022): 959–974. 35120736 10.1016/j.jhep.2021.12.022 PMC11090185

[28] M. Magaz , H. Giudicelli‐Lett , J. G. Abraldes , et al., “Porto‐Sinusoidal Vascular Liver Disorder With Portal Hypertension: Natural History and Long‐Term Outcome,” Journal of Hepatology 24 (2024): S0168–8278. 10.1016/j.jhep.2024.07.035 39181213

[29] B. Lausen and M. Schumacher , “Maximally Selected Rank Statistics,” Biometrics 48 (1992): 73–85.

[30] J. Evans , S. Newman , and S. Sherlock , “Liver Copper Levels in Intrahepatic Cholestasis of Childhood,” Gastroenterology 75, no. 5 (1978): 875–878. 700330

[31] P. Ferenci , G. Zollner , and M. Trauner , “Hepatic Transport Systems,” Journal of Gastroenterology and Hepatology 17, no. Suppl (2002): S105–S112. 12000597 10.1046/j.1440-1746.17.s1.15.x

[32] M. P. Salaspuro , P. Pikkarainen , P. Sipponen , E. Vuori , and T. A. Miettinen , “Hepatic Copper in Primary Biliary Cirrhosis: Biliary Excretion and Response to Penicillamine Treatment,” Gut 22, no. 11 (1981): 901–906. 7308845 10.1136/gut.22.11.901 PMC1419468

[33] R. L. Moss and L. A. Amii , “New Approaches to Understanding the Etiology and Treatment of Total Parenteral Nutrition‐Associated Cholestasis,” Seminars in Pediatric Surgery 8, no. 3 (1999): 140–147. 10461327 10.1016/s1055-8586(99)70015-6

[34] E. A. Roberts and M. L. Schilsky , “Current and Emerging Issues in Wilson's Disease,” New England Journal of Medicine 389, no. 10 (2023): 922–938. 37672695 10.1056/NEJMra1903585

[35] V. Hernández‐Gea , G. Campreciós , F. Betancourt , et al., “Co‐Expression Gene Network Analysis Reveals Novel Regulatory Pathways Involved in Porto‐Sinusoidal Vascular Disease,” Journal of Hepatology 75, no. 4 (2021): 924–934. 34052252 10.1016/j.jhep.2021.05.014

[36] J. Shan , A. Megarbane , A. Chouchane , et al., “Genetic Predisposition to Porto‐Sinusoidal Vascular Disorder: A Functional Genomic‐Based, Multigenerational Family Study,” Hepatology 77, no. 2 (2023): 501–511. 35989577 10.1002/hep.32735 PMC9869943

[37] G. Semmler , O. Petrenko , J. Jose Lozano , et al., “Metabolomic Profiles Differentiate Between Porto‐Sinusoidal Vascular Disorder, Liver Cirrhosis, and Healthy Individuals,” JHEP Reports. Published ahead of print. 10.1016/j.jhepr.2024.101208 PMC11609546 39624234

[38] D. Ozcelik , R. Ozaras , Z. Gurel , H. Uzun , and S. Aydin , “Copper‐Mediated Oxidative Stress in Rat Liver,” Biological Trace Element Research 96, no. 1–3 (2003): 209–215. 14716100 10.1385/BTER:96:1-3:209

[39] G. Faa , V. Nurchi , L. Demelia , et al., “Uneven Hepatic Copper Distribution in Wilson's Disease,” Journal of Hepatology 22, no. 3 (1995): 303–308. 7608481 10.1016/0168-8278(95)80283-5

[40] W. Osterode , G. Falkenberg , P. Ferenci , and F. Wrba , “Quantitative Trace Element Mapping in Liver Tissue From Patients With Wilson's Disease Determined by micro X‐Ray Fluorescence,” Journal of Trace Elements in Medicine and Biology 51 (2019): 42–49. 30466937 10.1016/j.jtemb.2018.09.007

