# Supplementary material for: Elevated Hepatic Copper Content in Porto‐Sinusoidal Vascular Disorder (PSVD): Leading Down a Wrong Track
Source: Liver Int. 2025 Jan 14;45(2):e16175. doi: 10.1111/liv.16175 (PMC11730389; doi:10.1111/liv.16175)
Supplement: Supplementary file 1 — Data S1. [file LIV-45-0-s001.docx]

**SUPPLEMENT**

## Supplementary Table 1

| *Patient characteristics* | | **PSVD cohort managed at our centre**  **n=136 (100%)** | **No evidence of cholestasis**  **n=90 (66%)** | **Any evidence of cholestasis**  **n=46 (34%)** | **p-value** |
| --- | --- | --- | --- | --- | --- |
| *Demographical data* | | | | | |
| Age, years, mean ± SD | | 47.6±15.8 | 46.0±16.0 | 50.6±15.1 | 0.107 |
| Body mass index, kg/m^2^ | | 25.1±5.0 | 25.5±4.7 | 24.3±5.4 | 0.187 |
| Sex, n (%) | | | | | |
|  | Male | 87 (64%) | 66 (73%) | 25 (54%) | **0.001** |
|  | Female | 49 (36%) | 24 (27%) | 21 (46%) |  |
| *Evidence of portal hypertension* | | | | | |
| Varices, n (%)* | | 73 (66%) | 44 (64%) | 29 (71%) | 0.455 |
| Splenomegaly, n (%)** | | 98 (78%) | 64 (76%) | 34 (81%) | 0.809 |
| Collaterals, n (%)** | | 78 (62%) | 46 (55%) | 32 (76%) | 0.085 |
| Portal vein thrombosis, n (%) | | 8 (6%) | 4 (4%) | 4 (9%) | 0.443 |
| Cavernoma, n (%) | | 18 (13%) | 11 (12%) | 7 (15%) | 0.626 |
| Presence of ascites, n (%) | | 32 (24%) | 18 (20%) | 14 (30%) | 0.144 |
| History of/current hepatic encephalopathy, n (%) | | 3 (2%) | 1 (1%) | 2 (4%) | 0.264 |
| History of variceal bleeding, n (%) | | 24 (18%) | 13 (14%) | 11 (24%) | 0.171 |
| HVPG, mmHg, mean ± SD | | 7±5 | 6±4 | 8±5 | **0.038** |
| Liver stiffness measurement, kPa, mean ± SD | | 10±7 | 9±7 | 11±7 | 0.135 |
| Continuous attenuation parameter, dB/m, mean ± SD | | 205±63 | 205±60 | 206±67 | 0.925 |
| *Diagnostic criteria of PSVD, n (%)* | | | | | |
|  | Specific clinical signs | 93 (68%) | 54 (60%) | 39 (85%) | **0.003** |
|  | Unspecific clinical signs | 130 (96%) | 87 (97%) | 43 (94%) | 0.406 |
|  | Specific histological signs | 93 (68%) | 65 (72%) | 28 (61%) | 0.178 |
|  | Unspecific histological signs | 116 (85%) | 79 (88%) | 37 (80%) | 0.253 |
| *Potential underlying factors, n (%)* | | | | | |
|  | HIV | 15 (11%) | 14 (16%) | 1 (2%) | **0.018** |
|  | History of specific medications | 52 (38%) | 33 (37%) | 19 (41%) | 0.599 |
|  | Associated comorbidities | 60 (44%) | 35 (39%) | 25 (54%) | 0.086 |
|  | Genetic disorders | 7 (5%) | 3 (3%) | 4 (9%) | 0.226 |
|  | Prothrombotic disorders | 20 (15%) | 13 (14%) | 7 (15%) | 0.904 |
| *Copper diagnostics, median (IQR) or mean ± SD* | | | | | |
|  | Hepatic copper content, µg/g | 30 (18-55) | 28 (16-55) | 34 (22-65) | 0.069 |
|  | Ceruloplasmin, mg/dL | 27±8 | 26±7 | 30±8 | **0.014** |
|  | Serum copper, µg/dL | 110 (85-136) | 109 (90-123) | 113 (93-130) | 0.695 |
| *Laboratory parameters, median (IQR) or mean ± SD* | | | | | |
|  | Platelet count, G/L | 132 (74-193) | 132 (70-187) | 126 (76-242) | 0.547 |
|  | Sodium, mmol/L | 139±3 | 139±3 | 139±3 | 0.594 |
|  | Creatinine, mg/dL | 0.8 (0.7-1.0) | 0.9 (0.7-1.0) | 0.7 (0.6-0.9) | **<0.001** |
|  | Albumin, g/L | 39.0±5.8 | 39.8±5.7 | 37.4±5.7 | **0.021** |
|  | Bilirubin, mg/dL | 0.8 (0.6-1.3) | 0.9 (0.6-1.3) | 0.8 (0.6-1.2) | 0.791 |
|  | AP, U/L | 88 (67-144) | 81 (60-104) | 156 (87-280) | **<0.001** |
|  | GGT, U/L | 60 (29-121) | 46 (22-93) | 100 (52-219) | **<0.001** |
|  | Bile acids, µmol/L | 9 (4-19) | 5 (2-7) | 19 (13-41) | **<0.001** |
|  | INR | 1.2±0.2 | 1.2±0.2 | 1.2±0.2 | 0.732 |
|  | von Willebrand factor antigen, % | 222±91 | 208±85 | 244±98 | **0.040** |
|  | AST, U/L | 36 (27-52) | 34 (26-47) | 41 (29-63) | **0.030** |
|  | ALT, U/L | 34 (22-57) | 34 (22-54) | 34 (24-79) | 0.242 |
|  | CRP, mg/dL | 0.3 (0.1-0.7) | 0.2 (0.1-0.6) | 0.4 (0.2-0.8) | 0.163 |
|  | Ammonia, mmol/L | 33 (25-42) | 31 (24-41) | 33 (26-45) | 0.572 |
|  | Hepatic iron content, µg/g | 397 (255-783) | 390 (212-800) | 420 (279-768) | 0.558 |
|  | Transferrin, mg/dL | 247±64 | 249±66 | 244±62 | 0.718 |
|  | Transferrin saturation | 17 (11-30) | 18 (12-30) | 16 (8-31) | 0.448 |
|  | Ferritin, µg/L | 79 (28-172) | 76 (29-148) | 97 (25-223) | 0.686 |

* data available in 81% of study cohort

** data available in 93% of study cohort

Categorical variables were reported as absolute (n) and relative frequencies (%), whereas continuous variables as mean ± SD or median (interquartile range [IQR]), as appropriate. Student’s t-test was used for group comparisons of normally distributed variables and Mann-Whitney-U-test for non-normally distributed variables. Group comparisons of categorical variables were performed using either Chi-squared or Fisher’s exact test, as appropriate. P-values in bold denote p<0.05.

**Supplementary Table 1.** Detailed patient characteristics at the time of diagnosis of PSVD of all patients with PSVD treated at the Medical University of Vienna

*Abbreviations: ALT alanine transaminase; AP alkaline phosphatase; AST aspartate transaminase; CRP C-reactive protein; GGT gamma-glutamyl transferase; HIV human immunodeficiency virus; HVPG hepatic venous pressure gradient; INR international normalized ratio; IQR interquartile range; n number; SD standard deviation*
